# Maximum Power Point Tracking Control for Non-Gaussian Wind Energy Conversion System by Using Survival Information Potential

**DOI:** 10.3390/e24060818

**Published:** 2022-06-11

**Authors:** Liping Yin, Lanlan Lai, Zhengju Zhu, Tao Li

**Affiliations:** 1 School of Ationautom, Nanjing University of Information Science & Technology, Nanjing 210044, China; lpyin@nuist.deu.cn (L.Y.); lanlan_lai@163.com (L.L.); zhuzhengju0426@163.com (Z.Z.); 2 Jiangsu Collaborative Innovation Center on Atmospheric Environment and Equipment Technology, Nanjing 210044, China

**Keywords:** wind energy conversion system, maximum power point tracking, stochastic distribution control, survival information potential

## Abstract

In this paper, a wind energy conversion system is studied to improve the conversion efficiency and maximize power output. Firstly, a nonlinear state space model is established with respect to shaft current, turbine rotational speed and power output in the wind energy conversion system. As the wind velocity can be descried as a non-Gaussian variable on the system model, the survival information potential is adopted to measure the uncertainty of the stochastic tracking error between the actual wind turbine rotation speed and the reference one. Secondly, to minimize the stochastic tracking error, the control input is obtained by recursively optimizing the performance index function which is constructed with consideration of both survival information potential and control input constraints. To avoid those complex probability formulation, a data driven method is adopted in the process of calculating the survival information potential. Finally, a simulation example is given to illustrate the efficiency of the proposed maximum power point tracking control method. The results demonstrate that by following this method, the actual wind turbine rotation speed can track the reference speed with less time, less overshoot and higher precision, and thus the power output can still be guaranteed under the influence of non-Gaussian wind noises.

## 1. Introduction

In recent years, new energy development has received extensive attention. Among all kinds of new energy, wind energy is the most widely used. However, the wind speed might be affected by factors such as seasonal changes, day-night alternation, and topography. For example, the average wind speed in spring and winter in the coastal areas of East China is usually larger than that in summer and autumn [1]. In those mountainous areas of southern China, due to irregular terrain and rough ground, it is more prone to occur a phenomenon called ‘airflow distortion’, and thus form blocking areas or leeward areas which result in uneven distribution of wind resources [2].

The output power of the wind turbine varies with the constantly changing wind speed, so the tip speed ratio will probably deviate from the optimal value, lower the utilization rate of wind energy and affect the service life of the wind turbine. Therefore, in order to improve the conversion efficiency of the wind energy conversion system, it is particularly important to carry out the maximum power tracking (MPPT) control for the wind energy conversion system [3,4]. In the existing literature about MPPT, output power is maximized by controlling the tip speed ratio according to the actual wind speed, and various control techniques have been applied to wind energy conversion system, such as PI control [5,6,7], sliding mode control [8,9,10]; predictive control [11,12,13]; robust control [14,15,16]; adaptive control [17,18,19] and LQG control [20,21,22] etc.

However, wind speed is actually a non-Gaussian random variable which usually leads to random fluctuation of output power in the wind energy conversion system. To minimize output power fluctuations and improve the energy conversion efficiency, we will remodel the system with full consideration of stochastic wind speed, output power of the wind turbine, as well as the dynamic subsystem of PMSG. The survival information potential (SIP) will also be adopted to describe the randomness of tracking error in this paper, and the optimal control law will be obtained by minimizing the SIP included performance index function.

## 2. System Description

**Figure 1:**
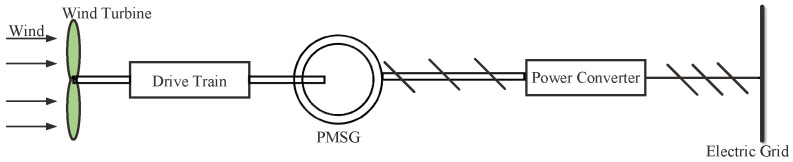
Schematic diagram of PMSG-based wind energy conversion system.

**Figure 2:**
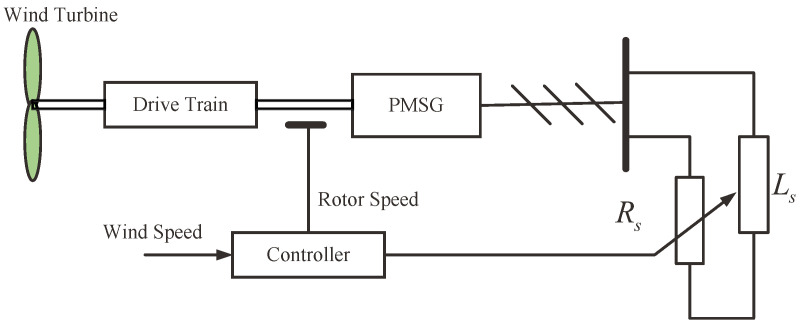
Equivalent schematic diagram of PMSG-based wind energy conversion system [26].

**Figure 3:**
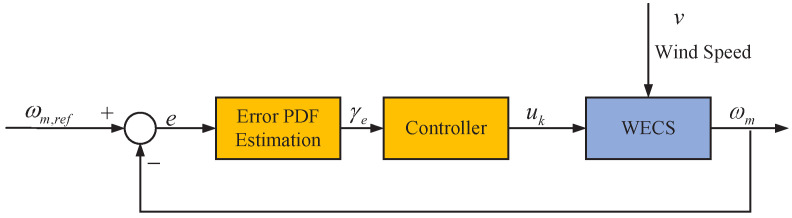
Block diagram of MPPT technology based on stochastic distribution control (SDC) strategy.

As shown in Figure 1, a wind energy conversion system usually includes a wind turbine, a drive train, permanent magnet synchronous generator (PMSG), power converter and electric grid constitute a PMSG-based wind energy conversion system. In the wind energy conversion system, the converter controls the rotation speed of the wind turbine through the voltage on the PMSG terminal, so as to control the power it generates. To establish a composite model for this conversion system, the wind turbine, the drive train and PMSG will be briefly introduced.

(A) Wind Turbine
  According to the characteristics of the wind turbine [23], the relationship between the wind speed *v* and output power $P_{w}$ can be expressed as follows:
  (1) $$\begin{array}{c} P_{w}=\frac{1}{2}C_{p}\left(\lambda \right)\rho \pi R_{t}^{2}v^{3} \end{array}$$
  where ρ is the air density, $R_{t}$ is the radius of the wind wheel and $C_{p}\left(\lambda \right)$ is the wind energy utilization coefficient. Usually, the conversion rate from wind’s kinetic energy to wind turbine’s mechanical energy is not 100%, so the coefficient $C_{p}\left(\lambda \right)$ is used here to describe the ability of wind turbines to convert wind energy. The larger $C_{p}\left(\lambda \right)$ is, the stronger ability the wind turbine will have.
  In fact, the wind energy utilization coefficient satisfies $C_{p}\left(\lambda \right)=\lambda C_{t}\left(\lambda \right)$ where $C_{t}\left(\lambda \right)$ is the wind turbine torque coefficient which can be approximated by a quadratic polynomial function with respect to the tip speed ratio λ [24] as follows
  (2) $$\begin{array}{c} C_{t}\left(\lambda \right)=\alpha_{0}+\alpha_{1}\lambda +\alpha_{2}\lambda^{2} \end{array}$$
  where $\alpha_{i}$ {i=0, 1, 2} is the coefficient of the quadratic polynomial. The tip speed ratio λ depends on the rotor speed $\omega_{m}$ of the wind turbine and the wind speed *v*, and the expression can be written as:
  (3) $$\begin{array}{c} \lambda =\frac{\omega_{m}R_{t}}{v} \end{array}$$
  Obviously, $\omega_{m}=\frac{\lambda v}{R_{t}}$, $P_{w}=\frac{1}{2}C_{p}\left(\lambda \right)\rho \pi R_{t}^{5}{\left(\frac{\omega_{m}}{\lambda }\right)}^{3}$.
(B) Drive Train
  The role of the drive train is to transfer the wind turbine mechanical torque $T_{m}$ to the PMSG. The kinematical equation of the drive train can be expressed as:
  (4) $$\begin{array}{c} J\frac{d\omega_{m}}{dt}=T_{m}-T_{g} \end{array}$$
  where $T_{m}$ is the torque of the wind turbine which can be expressed as:
  (5) $$\begin{array}{c} T_{m}=\frac{\rho \pi R_{t}^{3}v^{2}\left(a_{0}+a_{1}\lambda +a_{2}\lambda^{2}\right)}{2} \end{array}$$
  In (4), $T_{g}$ is the electromagnetic torque of the PMSG, *J* is the inertia of the rotating part, and $\omega_{m}$ is the rotor speed of the wind turbine.
(C) PMSG
  The stator voltages of the PMSG in the d-q frame can be expressed as [25]:
  (6) $$\begin{array}{c} L_{d}\frac{di_{d}}{dt}=-Ri_{d}+\omega_{e}L_{q}i_{q}-u_{d} \end{array}$$
  (7) $$\begin{array}{c} L_{q}\frac{di_{q}}{dt}=-Ri_{q}-\omega_{e}L_{d}i_{d}+\omega_{e}\phi_{m}-u_{q} \end{array}$$
  where $u_{d}$ and $u_{q}$ are the *d* shaft voltage and *q* shaft voltage in the rotor coordinate system, respectively; $i_{d}$ and $i_{q}$ are the *d* shaft current and *q* shaft current in the rotor coordinate system, respectively; *R* is the stator resistance; $L_{d}$ and $L_{q}$ are the inductance in the *d*-*q* coordinate system, $\omega_{e}=p\omega_{m}$ is the electrical angular velocity of the generator; *p* is the number of pole pairs of the PMSG; and $\phi_{m}$ is the flux that is constant due to permanent magnets.
(D) Power Converter and Electric Grid
  The job that the power converter does can be divided into three steps: It firstly converts AC voltage from PMSG to DC voltage, then converts DC voltage back to AC but variable voltage, and finally, it puts variable AC voltage into the grid.
  The wind energy conversion system has partial load mode and full load mode. When the wind speed is lower than the rated wind speed, the wind energy conversion system operates in the partial load mode. When the wind speed is higher than the rated wind speed, the wind energy conversion system operates in the full mode. To explicitly explain our algorithm, we will only consider about the partial load mode. For full load mode, the research method will be quite similarly.
  In partial load mode, the power electronics dynamic is neglected because it is significantly more rapid than the PMSG-based wind energy conversion system dynamic. As shown in Figure 2, the power converter and the electric grid are equivalent to a parallel connection of a constant value inductance $L_{s}$ and a variable resistance $R_{s}$, and thus in this paper, it is regarded as the equivalent load of the PMSG. In Figure 2, the resistance value of $R_{s}$ changes with duty ratio of the control pulse of the power converter.
  According to the Figure 2 and literature [26], the stator voltages of the PMSG equivalent model can be formulated as:
  (8) $$\begin{array}{c} \left(L_{d}+L_{s}\right)\frac{di_{d}}{dt}=-\left(R+R_{s}\right)i_{d}+p\left(L_{q}-L_{s}\right)i_{q}\omega_{m} \end{array}$$
  (9) $$\begin{array}{c} \left(L_{q}+L_{s}\right)\frac{di_{q}}{dt}=-\left(R+R_{s}\right)i_{q}-p\left(L_{d}+L_{s}\right)i_{d}\omega_{m}+p\omega_{m}\phi_{m} \end{array}$$
  On the other hand, the electromagnetic torque $T_{g}$ in (4) can be expressed as:
  (10) $$\begin{array}{c} T_{g}=p\phi_{m}i_{q}+p\left(L_{d}-L_{q}\right)i_{d}i_{q} \end{array}$$
  In order to simplify the system model, assuming that $L_{d}=L_{q}$ [27], then the electromagnetic torque $T_{g}$ can be further written as:
  (11) $$\begin{array}{c} T_{g}=p\phi_{m}i_{q} \end{array}$$
  Substituting (5) and (11) into (4), the dynamic equation of wind turbine speed $\omega_{m}$ can be written as:
  (12) $$\begin{array}{c} \frac{d\omega_{m}}{dt}=\frac{1}{J}\left(\frac{\rho \pi R_{t}^{3}v^{2}\left(a_{0}+a_{1}\lambda +a_{2}\lambda^{2}\right)}{2}-p\phi_{m}i_{q}\right) \end{array}$$
  Let $x={\left[x_{1},x_{2},x_{3}\right]}^{T}$ = ${\left[i_{d},i_{q},\omega_{m}\right]}^{T}$, $u=R_{s}$. Combining (8), (9) and (12), we can get the following nonlinear state space model:
  (13) $$\begin{array}{c} \left\{\begin{array}{c} \dot{x}=f\left(x,v\right)+g\left(x\right)u\\ y=h(x) \end{array}\right. \end{array}$$
  where $u=R_{s}$ is the control variable, and the detailed expression of f(x), g(x) and h(x) are shown as follows
  (14) $$\begin{array}{c} f\left(x\right)=\left[\begin{array}{c} -\frac{R}{L_{d}+L_{s}}x_{1}+p\frac{L_{q}-L_{s}}{L_{d}+L_{s}}x_{2}x_{3}\\ -\frac{R}{L_{q}+L_{s}}x_{2}-p\left(L_{d}+L_{s}\right)x_{1}x_{3}+p\phi_{m}x_{3}\\ \frac{1}{J}\left(-p\phi_{m}x_{2}+d_{1}v^{2}+d_{2}vx_{3}+d_{3}{x_{3}}^{2}\right) \end{array}\right] \end{array}$$
  (15) $$\begin{array}{c} g\left(x\right)=\left[\begin{array}{ccc} -\frac{1}{L_{d}+L_{s}}x_{1} & -\frac{1}{L_{q}+L_{s}}x_{2} & 0 \end{array}\right] \end{array}$$
  (16) $$\begin{array}{c} h\left(x\right)=\frac{1}{2}C_{p}\left(\lambda \right)\rho \pi R_{t}^{5}{\left(\frac{x_{3}}{\lambda }\right)}^{3} \end{array}$$
  In (12), the output *y* means the output power $P_{w}$ in the wind energy conversion system, and $d_{1}=\frac{1}{2}\pi \rho R_{t}^{3}\alpha_{0}$, $d_{2}=\frac{1}{2}\pi \rho R_{t}^{4}\alpha_{1}$, $d_{3}=\frac{1}{2}\pi \rho R_{t}^{5}\alpha_{2}$ are all known coefficients.
  According to (16), the output power $P_{w}$(y) increases monotonically with the wind turbine rotational speed $\omega_{m}$$\left(x_{3}\right)$. If the optimal rotational speed $\omega_{m}$ can track $\omega_{m,ref}$, the output power will reach the maximum. Therefore, the output power control of the wind power generation system in the partial load mode can be turned into the control of the wind turbine speed $\omega_{m}$.
  From the above analysis, it can be seen that the ability of the wind turbine to capture the maximum wind energy is equivalent to controlling the rotational speed of the wind turbine to track the optimal rotational speed. As the wind speed is usually a non-Gaussian random variable, the control theory using only mean or variance is not sufficient.
  In fact, due to the influence of non-Gaussian random variable *v*, the wind turbine speed $\omega_{m}$ is also a non-Gaussian random variable. Denote the tracking error as
  (17) $$\begin{array}{c} e=\omega_{m,ref}-\omega_{m}. \end{array}$$
  where $\omega_{m,ref}$ is target value. As shown in Figure 3, our objective is to design a controller to make the probability density function (PDF) of power tracking error *e* get close to an impulse shaped PDF with mean value 0.

## 3. Controller Design

In this section, the SIP will be used to measure the uncertainty of the stochastic tracking error $e_{k}$. The SIP is a little similar to traditional entropy, but it overcomes the property of traditional entropy that it has no translation invariance (that is, it changes with the change of distribution). And it is easy to compute the actual value from the sample data to avoid selecting kernel width and computing kernel [28,29]. Based on SIP, the control input can be obtained by recursively optimizing the performance index function.

### 3.1. Performance Index Function

System (13) has single input and single output. Recalling $e=\omega_{m,ref}-\omega_{m}$ in (17), the α order survival information potential of *e* can be defined follows:

(18) $$\begin{array}{c} S_{\alpha }\left(e\right)=\int_{R_{+}}{\bar{F}}_{\left|e\right|}^{\alpha }\left(e\right)de\left(\alpha >0\right) \end{array}$$

where ${\bar{F}}_{\left|e\right|}\left(x\right)$ = $P(\left|e\right|>x)$ = $E[I\left(\left|e\right|>x\right)]$ is the survival function of the random vector $\left|e\right|$, $e\in R_{+}$, *I* is the indicator function. In this paper, the *k* time performance indicator function is selected as follows:

(19) $$\begin{array}{c} J=R_{1}S_{\alpha }\left(e_{k}\right)+\frac{1}{2}u_{k}^{T}R_{2}u_{k} \end{array}$$

where $R_{1}$ and $R_{2}$ are constant weights, $\frac{1}{2}u_{k}^{T}R_{2}u_{k}$ represents the constraints of the control input, and $S_{\alpha }\left(e_{k}\right)$ represents the survival information potential of the tracking error.

### 3.2. SIP Estimation for Tracking Error

The probability distribution of the error is usually unknown. Therefore, here we adopt a data-driven approach to estimate the SIP of the tracking error [30,31]. Supposing at time k there exist error samples $(e_{1,k},e_{2,k},\cdots ,e_{N,k})$ which represent a set of *N* independent and identically distributed samples for *e*, then the estimated value of the survival function ${\hat{\bar{F}}}_{e_{k}}\left(x\right)$ can be expressed as:

(20) $$\begin{array}{c} {\hat{\bar{F}}}_{e_{k}}\left(x\right)=\frac{1}{N}\overset{N}{\underset{i=1}{\sum }}I\left(e_{k,i}>x\right) \end{array}$$

Furthermore, the sliding window method will be used to estimate the SIP of the tracking error $e_{k}$. Let *N* be sliding window width. For the sample sequence $(e_{1,k},e_{2,k},\cdots ,e_{N,k})$, and without the loss of generality, supposing that $0\leq \left|e_{k,1}\right|\leq \left|e_{k,2}\right|\leq \cdots \leq \left|e_{k,N}\right|$, the SIP estimate of the tracking error $e_{k}$ can be further expressed as:

(21) $$\begin{array}{c} \begin{array}{c} {\hat{S}}_{\alpha }\left(e_{k}\right)=\int_{0}^{\infty }{\left(\frac{1}{N}\overset{N}{\underset{i=1}{\sum }}I\left(\left|e_{k,i}\right|>e\right)\right)}^{\alpha }de=\overset{N}{\underset{j=1}{\sum }}{\int_{\left|e_{k,j}-1\right|}^{\left|e_{k,j}\right|}\left(\frac{1}{N}\overset{N}{\underset{i=1}{\sum }}I\left(\left|e_{k,i}\right|>e\right)\right)}^{\alpha }de\\ =\overset{N}{\underset{j=1}{\sum }}{\left(\frac{N-j+1}{N}\right)}^{\alpha }\left(\left|e_{k,j}\right|-\left|e_{k,j-1}\right|\right) \end{array} \end{array}$$

where $e_{k,0}=0$, then (21) can be simplified as:

(22) $$\begin{array}{c} \begin{array}{c} {\hat{S}}_{\alpha }\left(e_{k}\right)=\overset{N}{\underset{j=1}{\sum }}{\left(\frac{N-j+1}{N}\right)}^{\alpha }\left(\left|e_{k,j}\right|-\left|e_{k,j-1}\right|\right)=\left(1-{\left(\frac{N-1}{N}\right)}^{\alpha }\right)\left|e_{k,1}\right|\\ +\cdots +\left({\left(\frac{2}{N}\right)}^{\alpha }-{\left(\frac{1}{N}\right)}^{\alpha }\right)\left|e_{k,N-1}\right|+{\left(\frac{1}{N}\right)}^{\alpha }\left|e_{k,N}\right|\\ =\overset{N}{\underset{j=1}{\sum }}\mu_{j}\left|e_{k,j}\right| \end{array} \end{array}$$

In (22), the weight $\mu_{j}={\left(\frac{N-j+1}{N}\right)}^{\alpha }-{\left(\frac{N-j}{N}\right)}^{\alpha }$, $\mu_{j}\left(j=1,2,\cdots ,N\right)$ depends on the number of samples *N*. Obviously $\mu_{j}\geq 0,\overset{N}{\underset{j=1}{\sum }}\mu_{j}=1$.

From (22) it can be known that the SIP estimation of *e* is the weighted sum of $e_{k,j}$. Actually, the SIP is not smooth when $e_{k,j}=0$. To overcome this, we consider the SIP of squared $e_{k}$, and then Equation (22) can be converted to the following

(23) $$\begin{array}{c} {\hat{S}}_{\alpha }\left(e_{k}^{2}\right)=\overset{N}{\underset{j=1}{\sum }}\mu_{j}e_{k,j}^{2} \end{array}$$

Furthermore, the performance index function (19) can be transformed into

(24) $$\begin{array}{c} J=R_{1}{\hat{S}}_{\alpha }\left(e_{k}^{2}\right)+\frac{1}{2}u_{k}^{T}R_{2}u_{k} \end{array}$$

For convenience, we denote the first term on the right side of Equation (24) as:

(25) $$\begin{array}{c} \bar{J}=R_{1}{\hat{S}}_{\alpha }\left(e_{k}^{2}\right) \end{array}$$

### 3.3. Optimal Control Law

In this paper, *J* in Equation (24) is the performance index to be optimized. The control objective is then transformed into finding the control input $u_{k}^{*}$, so that *J* is minimized at each sample time *k*, that is,

(26) $$\begin{array}{c} u_{k}^{*}=\arg\min J=\arg\min\left(R_{1}{\hat{S}}_{\alpha }\left(e_{k}^{2}\right)+\frac{1}{2}u_{k}^{T}R_{2}u_{k}\right) \end{array}$$

Note $u_{k}=u_{k-1}+\Delta u_{k}$, $\Theta_{k}\left(u_{k}\right)={\hat{S}}_{\alpha }\left(e_{k}^{2}\right)$. Take the Taylor expansion of $\Theta_{k}\left(u_{k}\right)$ at $u_{k-1}$, we obtain:

(27) $$\begin{array}{c} \Theta_{k}\left(u_{k}\right)\approx \Theta_{k}\left(u_{k-1}\right)+\frac{\partial \Theta_{k}\left(u_{k-1}\right)}{\partial u_{k-1}}\left(u_{k}-u_{k-1}\right)+\frac{1}{2}\Theta_{k2}\frac{\partial^{2}\Theta_{k}\left(u_{k-1}\right)}{\partial^{2}u_{k-1}}{\left(u_{k}-u_{k-1}\right)}^{2} \end{array}$$

(28) $$\begin{array}{c} \Theta_{k}\left(u_{k}\right)=\Theta_{k,0}+\Theta_{k,1}\Delta u_{k}+\frac{1}{2}\Theta_{k,2}\Delta u_{k}^{2} \end{array}$$

Substituting (28) into (24) yields:

(29) $$\begin{array}{c} u_{k}^{*}=u_{k-1}+\Delta u_{k}^{*}=u_{k-1}-{\left(R_{1}\Theta_{k,2}+R_{2}\right)}^{-1}\left(R_{1}\Theta_{k,1}+R_{2}u_{k-1}\right) \end{array}$$

Then the optimal control input $u_{k}^{*}$ can be calculated by the following equation

(30) $$\begin{array}{c} \frac{\partial J(u_{k})}{\partial \Delta u_{k}}=0 \end{array}$$

From (29) and (30), $u_{k}^{*}$ can be calculated that:

(31) $$\begin{array}{c} u_{k}^{*}=u_{k-1}+\Delta u_{k}^{*}=u_{k-1}-{\left(R_{1}\Theta_{k,2}+R_{2}\right)}^{-1}\left(R_{1}\Theta_{k,1}+R_{2}u_{k-1}\right) \end{array}$$

where the weights satisfy $R_{1}$ > 0, $R_{2}$ > 0, and $R_{1}\Theta_{k,2}+R_{2}>0$ should be satisfied.

The algorithm of the optimal control law can be summarized as follows:

Step 1: Initialize control input $u_{0}$;
Step 2: Select the sliding window width *N*, the value of the SIP order α and the weight $R_{1}$ and $R_{2}$ in Equation (24);
Step 3: Calculate the SIP and the performance index value $\bar{J}$ by Equations (23) and (24) respectively;
Step 4: Calculate the $\Theta_{k,1}$ and $\Theta_{k,2}$ by Equation (28);
Step 5: Solve the optimal control input $u_{k}$ by Equation (31);
Step 6: According to $u_{k}=u_{k-1}+\Delta u_{k}$, update control law;
Step 7: Increase *k* by 1 to repeat the process from the step 3 to the step 6.

## 4. Simulation

In this section, the performance of the proposed control strategy will be verified and compared with those results in [6,32].

In the performance index *J*, the weight is α=2, $R_{1}=1$, $R_{2}=0.0001$ and the width of the sliding window is N=100. The sampling interval is chosen as 0.1 s. For (13), we use the same parameters as in [26], that is R=3.3 Ω, $L_{d}=l_{q}=41.56$ mH, $L_{s}=0.1452$ H, p=3, $R_{t}=2.5$ m, $\phi_{m}=0.4382$ Wb, $\alpha_{0}=0.1253$, $\alpha_{1}=-0.0470.1253$, $\alpha_{2}=-0.005$.

The basic wind speed model can be approximately determined by the Weibull distribution parameters obtained from wind measurements in the wind farm as [33]:

(32) $$\begin{array}{c} v_{b}=C•\Gamma \left(1+\frac{1}{K}\right) \end{array}$$

where C,K are the scale and shape parameters of the Weibull distribution, respectively. Γ is the gamma function. In this simulation experiment, the basic wind speed is taken as a fixed constant *k*, namely

(33) $$\begin{array}{c} v_{b}=k\;m/s \end{array}$$

and *k* is selected to be 7. Actually, the wind speed changes irregularly. This irregular wind speed variation can be described by a stochastic wind model as:

(34) $$\begin{array}{c} v_{s}=v_{s\max}\cdot rand\left(-1,1\right)\cdot \cos\left(\omega_{s}t+\varphi \right) \end{array}$$

where $v_{s}$ is random wind; $v_{s\max}$ is the maximum value of random wind speed; $\omega_{s}\in \left\{0.5-2\pi \;\mathrm{rad}/s\right\}$ is the average distance of wind speed fluctuation; φ∈{0−2π} is a random variable obeying uniform distribution.

**Figure 4:**
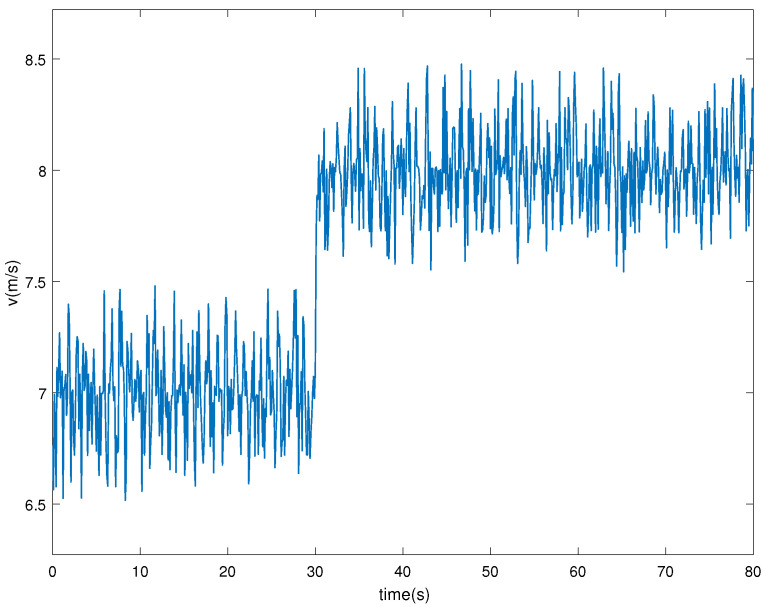
The combined wind speed.

The wind energy conversion system operates with a combination of base wind speed $v_{b}=7$ m/s and random wind speeds $v_{s}$ initially, that is $v=v_{b}+v_{s}$, and thus *v* is non-Gaussian. After 30 s, the basic wind speed becomes 8 m/s, as shown in Figure 4.

**Figure 5:**
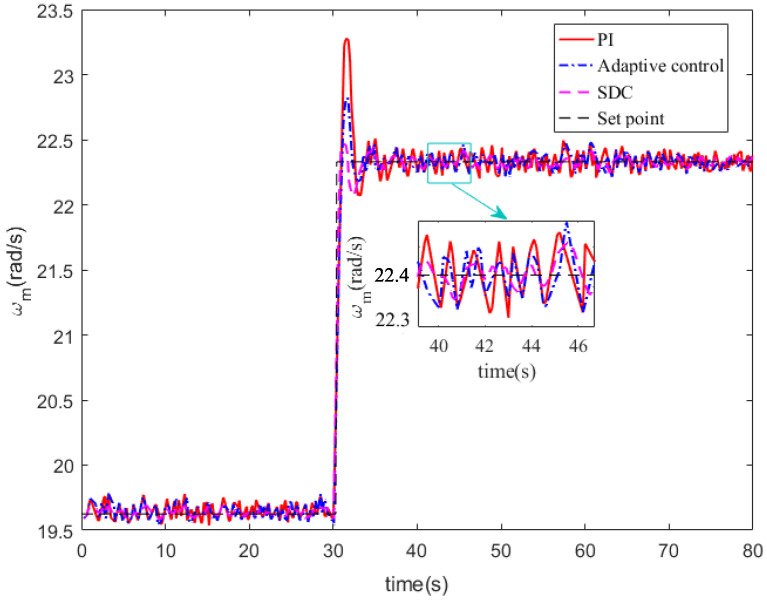
The rotor speed $\omega_{m}$.

Figure 5 is the rotor speed $w_{m}$ from which we can see that although the wind speed is changing, after the stochastic control strategy, the rotor speed can still track the taget rotor speed. Compared with PI control, the overshoot under SDC is smaller and the tracking time SDC needs is shorter.

**Figure 6:**
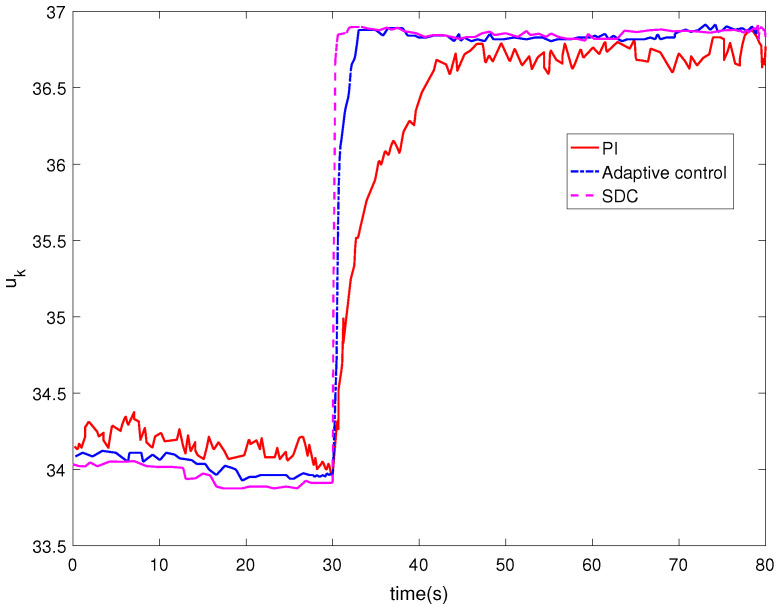
The control input $u_{k}$.

**Figure 7:**
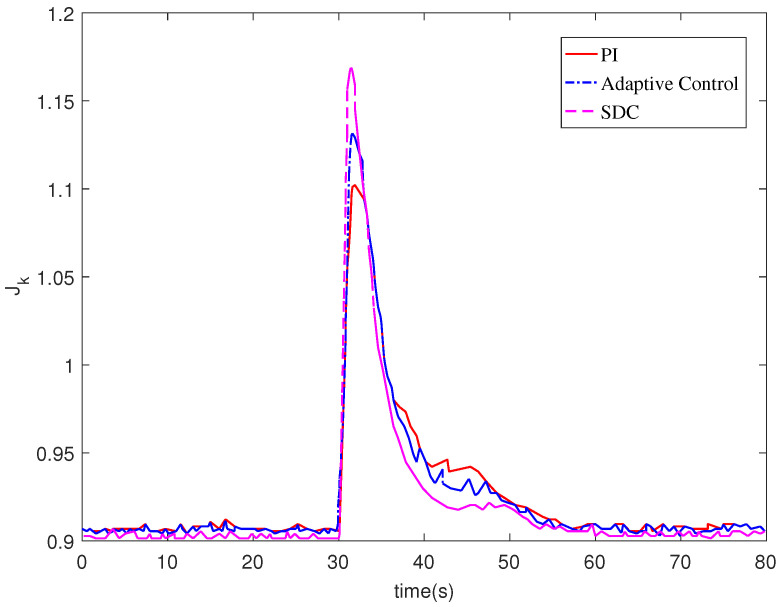
Performance index function $J_{k}$.

Figure 6 shows the control input. It can be seen from Figure 6 that no matter which control method is used, the system input will eventually tend to a stable value, but the input of the controller designed in this paper can tend to be stable in a shorter time. As the performance index function, we can see from Figure 7 that the change for the performance index generally decreases gradually and eventually tends to be stable.

**Figure 8:**
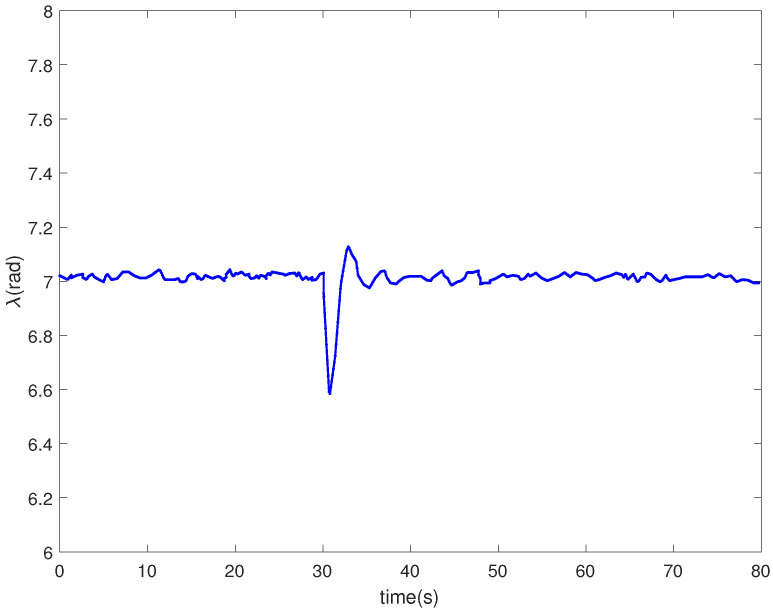
Tip speed ratio of a wind turbine.

**Figure 9:**
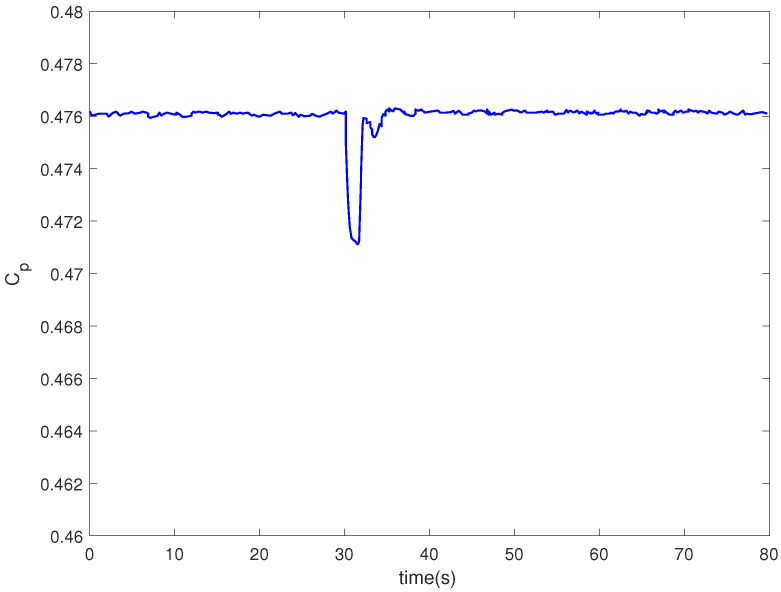
Power coefficient of a wind turbine.

Figure 8 is the tip speed ratio. As shown in Figure 8, the tip speed ratio varies in the range of [6.5, 7.2]. The wind energy utilization coefficient is kept in the range of [0.471, 0.476]. In the vicinity of the optimal tip speed ratio $\lambda_{opt}$, the utilization rate of wind energy is higher, as shown in Figure 9.

**Figure 10:**
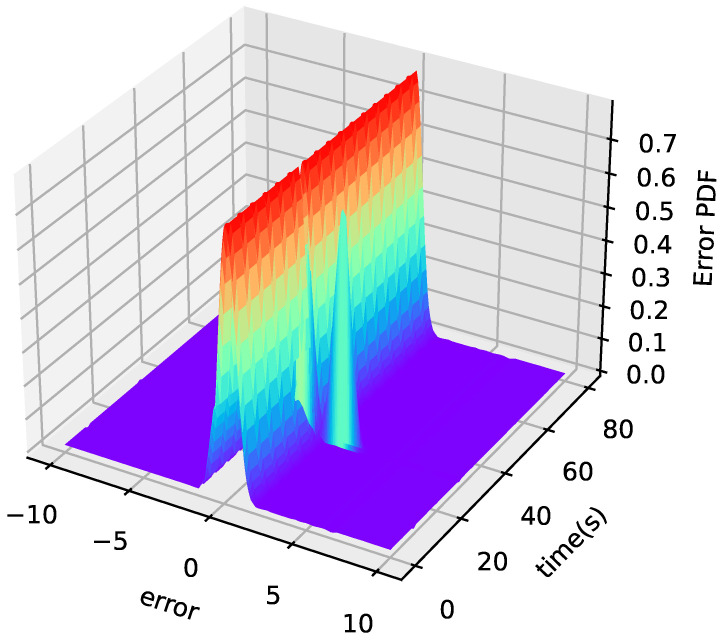
3D mesh plot of the tracking error PDF.

**Figure 11:**
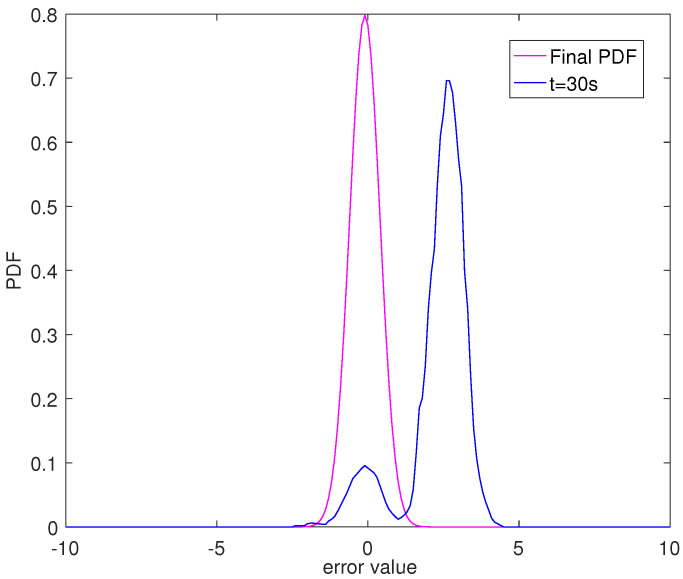
Tracking error PDF at different sample times.

The 3D PDF of tracking error *e* is shown in Figure 10. Figure 11 is the PDF of the tracking error at t=30 s. As shown in Figure 10, the PDF shape of the tracking error becomes narrower and sharper over time. This shows that the method proposed in this paper can effectively reduce the influence of randomness on the system.

## 5. Conclusions and Future Work

This paper studies the MPPT control strategy for wind energy conversion system. Firstly, the model of the composite wind energy conversion system is established. Then, for the non-Gaussian wind energy conversion system, a MPPT control method based on the SDC strategy is designed, and the performance index function is established based on the survival information potential. The optimal control law is obtained by minimizing the performance index function. The experimental results show that, compared with the traditional PI control or adaptive control, the proposed stochastic distribution controller can provide smoother rotor speed regulation and avoid unnecessary power fluctuations in the presence of stochastic wind speed. The small fluctuation of wind energy utilization coefficient further shows that the control algorithm can ensure MPPT of the wind energy conversion system.

Further research includes study of MPPT control at full load model. The stability or convergence analysis of the control algorithm is quite challenging and deserves attention.

## Data Availability

Not applicable.

## References

[1] Feng S., Gong D., Zhang Z., He X., Guo D. (2009). Wind-Chill temperature changes in winter over China during the last 50 years. Acta Geogr. Sinica.

[2] Li Y., Wu X., Li Q., Tee K.F. (2018). Assessment of onshore wind energy potential under different geographical climate conditions in China. Energy.

[3] Yang B., Zhang X., Tao Y., Shu H. (2017). Grouped grey wolf optimizer for maximum power point tracking of doubly-fed induction generator based wind turbine. Energy Convers. Manag..

[4] Koutroulis E., Kalaitzakis K. (2006). Design of a maximum power tracking system for wind-energy-conversion applications. IEEE Trans. Ind. Electron..

[5] Rachid E., Ahmed A.D., Mahdi D. (2018). A novel design of PI current controller for PMSG-based wind turbine considering transient performance specifications and control saturation. IEEE Trans. Ind. Electron..

[6] Wang J.S., Tse N., Gao Z.W. (2011). Synthesis on PI-based pitch controller of large wind turbines generator. Energy Convers. Manag..

[7] José G.H., Rubén S.C., Roberto V.B. (2021). A novel MPPT PI discrete reverse-acting controller for a wind energy conversion system. Renew. Energy.

[8] Rhaili S.E., Abbou A., Marhraoui S., Moutchou R. (2020). Robust sliding mode control with five sliding surfaces of five-phase PMSG based variable speed wind energy conversion system. Renew. Energy.

[9] Chojaa H., Derouich A., Chehaidia S.E. (2021). Integral sliding mode control for DFIG based WECS with MPPT based on artificial neural network under a real wind profile. Energy Rep..

[10] Darkhan Z., Matteo R., Ton D.D. (2022). Adaptive super-twisting sliding mode control for maximum power point tracking of PMSG-based wind energy conversion systems. Renew. Energy.

[11] Atif I., Deng Y., Adeel S. (2020). Efficacious pitch angle control of variable-speed wind turbine using fuzzy based predictive controller. Energy Rep..

[12] Riad A., Toufik R., Djamila R., Abdelmounaim T. (2016). Application of nonlinear predictive control for charging the battery using wind energy with permanent magnet synchronous generator. Int. J. Hydrog. Energy.

[13] Lin Z., Chen Z., Liu J. (2019). Coordinated mechanical loads and power optimization of wind energy conversion systems with variable-weight model predictive control strategy. Appl. Energy.

[14] Satyajit D., Bidyadhar S. (2018). A *H*_∞_ Robust active and reactive power control scheme for a PMSG-based wind energy conversion system. IEEE Trans. Energy Convers..

[15] Hadi D., Amir V. Robust control of a permanent magnet synchronous generators based wind energy conversion. Proceedings of the 2021 7th International Conference on Control, Instrumentation and Automation (ICCIA).

[16] Amina M., Sandrine L.B., Helmi A. (2019). Robust control of a wind conversion system based on a hybrid excitation synchronous generator: A comparison between *H*_∞_ and CRONE controllers. Math. Comput. Simulat..

[17] Hui J., Bakhshai A. A new adaptive control algorithm for maximum power point tracking for wind energy conversion systems. Proceedings of the 2008 IEEE Power Electronics Specialists Conference.

[18] Chen J., Yao W., Zhang C., Ren Y., Jiang L. (2019). Design of robust MPPT controller for grid-connected PMSG-based wind turbine via perturbation observation based nonlinear adaptive control. Renew. Energy.

[19] Aubrée R., Auger F., Macé M., Loron L. (2016). Design of an efficient small wind-energy conversion system with an adaptive sensorless MPPT strategy. Renew. Energy.

[20] Munteanu I., Cutululis N.A., Antoneta I.B. (2005). Optimization of variable speed wind power systems based on a LQG approach. Control Eeg. Pract..

[21] Lescher F., Zhao J., Martinez A. (2005). LQG multiple model control of a variable speed, pitch regulated wind turbine. Chalmers.

[22] Ronilson R. (2011). A sensorless control for a variable speed wind turbine operating at partial load. Renew. Energy.

[23] Uehara A., Pratap A., Goya T., Senjyu T., Yona A. (2011). A coordinated control method to smooth wind power fluctuations of a PMSG-based WECS. IEEE Trans. Energy Convers..

[24] Hur S., Leithead W.E. (2017). Model predictive and linear quadratic Gaussian control of a wind turbine. Optim. Contr. Appl. Met..

[25] Tan K., Islam S. (2004). Optimum control strategies in energy conversion of PMSG wind turbine system without mechanical sensors. IEEE Trans. Energy Convers..

[26] Munteanur I.A., Cutululis N.A., Bratcu A.I. (2008). Optimal Control of Wind Energy Systems.

[27] Wang W., Wu D.H., Wang Y. *H*_∞_ gain scheduling control of PMSG-based wind power conversion system. Proceedings of the 2010 5th IEEE Conference on Industrial Electronics and Applications.

[28] Zhang Q., Wang H. (2022). A novel data-based stochastic distribution control for Non-Gaussian stochastic systems. IEEE Trans. Automat. Contr..

[29] Yin L., Wang H., Guo L., Zhang H. (2021). Data-driven pareto-DE-based intelligent optimal operational control for stochastic processes. IEEE Trans. Syst. Man Cybern. Syst..

[30] Chen B., Zhu P., Principe J.C. (2012). Survival Information Potential: A new criterion for adaptive system training. IEEE Trans. Signal Process..

[31] Ren M.F., Cheng T., Chen J.H., Xu X.Y., Cheng L. (2016). Single neuron stochastic predictive PID control algorithm for nonlinear and Non-Gaussian systems using the survival information potential criterion. Entropy.

[32] Abdelhameed E.H., Ahmed H.H. Adaptive maximum power tracking control technique for wind energy conversion systems. Proceedings of the 2018 Twentieth International Middle East Power Systems Conference (MEPCON).

[33] Chang T.P. (2011). Performance comparison of six numerical methods in estimating Weibull parameters for wind energy application. Appl. Energy.

